# Youths with asthma and their experiences of self‐management education: A systematic review of qualitative evidence

**DOI:** 10.1111/jan.15459

**Published:** 2022-10-14

**Authors:** Karen McTague, Geraldine Prizeman, Stephen Shelly, Jessica Eustace‐Cook, Edward McCann

**Affiliations:** ^1^ School of Nursing and Midwifery Trinity College Dublin Ireland; ^2^ Trinity Centre for Practice and Healthcare Innovation School of Nursing and Midwifery, Trinity College Dublin Ireland; ^3^ Department of Respiratory St James's Hospital Dublin Ireland; ^4^ The Library of Trinity College Dublin Trinity College Dublin Ireland; ^5^ School of Health and Psychology City University of London London UK

**Keywords:** asthma, education, nurse, qualitative research, self‐management, systematic literature review, youth; adolescents

## Abstract

**Aims:**

To identify and synthesize the available evidence of youths with asthma and their experience of self‐management education.

**Design:**

Systematic literature review of qualitative studies with meta‐synthesis of findings.

**Data sources:**

We searched five databases, CINAHL Complete, Embase, MEDLINE (EBSCO) PsycINFO, ASSIA and the Global Index Medicus (formerly the WHOLIS). Initial search in September 2019 and updated in July 2020 and July 2022.

**Review Methods:**

The systematic review was conducted in accordance with the JBI methodology for systematic reviews of qualitative evidence. Qualitative data were extracted, meta‐summarized and then meta‐synthesized.

**Results:**

Eighteen studies were identified for inclusion in this review and three themes were identified: The theory and practice gap, contemporary health‐seeking preferences and the psychosocial impacts of living with asthma.

**Conclusion:**

The needs of youths with asthma are specific and must be measurable against the change in asthma outcomes for this group. They have unmet self‐management educational needs that stakeholders, involved in their care and support, should address. Education and practice policy should focus on youth‐centric approaches. Through meaningful engagement with youths, stakeholders can identify their support needs, requirements and preferences to successfully underpin the theory and practice of self‐management education.

**Impact:**

This review synthesized evidence of youths with asthma and their experiences of self‐management education, highlighting their specific self‐management information needs. The findings highlight several implications for healthcare professionals in education, practice and research. This age profile is under‐explored and further research into this population would work towards filling the theory and practice gap and highlighting the identified psychosocial issues faced by this group.

## 1 INTRODUCTION AND BACKGROUND

Figures from the World Health Organization (WHO) estimate that 262 million people are affected by asthma (WHO, 2021). Youths are a subset of the healthy population that are affected by chronic diseases such as asthma. Despite the abundance of educational resources for asthma patients, asthma self‐management is unattainable for some youths (Rhee et al., 2009). There are many reasons cited in the literature, including poor adherence to treatment plans including medication management, issues of developmental maturity and the perceived willingness to take responsibility for their actions and the resultant consequences of those actions, that could impact on their ability to maintain asthma control (Kime et al., 2013; Rhee et al., 2009). Bobbit (1961) describes cognitive maturity as the psychological development that entails an awareness and insight into the cause and effect of actions. Global health policy mandates the reduction in the continuum of asthma between morbidity and mortality of this chronic disease (GINA, 2021). However, positioning global asthma self‐management goals for youths requires healthcare stakeholders to reflect on the evidence of youth asthma self‐management experiences. In acknowledging their experience of asthma education, only then can all stakeholders, healthcare professionals, parents and youths, recognize and work towards the evolving requirements of youth‐specific asthma self‐management and negate poor clinical outcomes.

Asthma is a respiratory condition of complex physiological interactions compounded by the unpredictability of asthma symptoms. Characteristics of the disease are distinguishable by symptoms of wheeze, shortness of breath and production of mucus (Quirt et al., 2018). The spectrum of physical symptoms can be mild and managed in accordance with asthma action plans or severe symptoms, requiring hospital attendance with the goal of asthma control. Unfortunately, asthma deaths are reported, thus highlighting the unpredictable nature of asthma that does not discriminate by age (Global Asthma Network, 2018). Therefore, asthma self‐management education is the cornerstone to negate poor youth outcomes. Self‐management education provides patients with the tools to make informed choices about their chronic disease diagnosis to enable quality of living (Kime et al., 2013; Bodenheimer et al., 2002). Understanding an asthma diagnosis includes education on the condition, medication information, identifying triggers as well as avoidance strategies and personal commitment, all of which are key to self‐management success. However, Kime et al. (2013) argue that the adoption of adult models of self‐management education, designed and developed by healthcare practitioners, make assumption of the self‐management needs of the recipients rather than expressed needs of young people.

Against this backdrop of personal commitment of asthma self‐management concerns arise for the youth cohort with a diagnosis of asthma. Youthhood is a period of discovery, curiosity and development in personal relationships and social interactions, that can impact positively or negatively in decision making about asthma management (Naimi et al., 2009). Furthermore, poor decisions result in consequences as significant as death (Uzuncakmak & Beser, 2017). However, some youths recognize life‐threatening events as the precursor to health‐seeking behaviours in respect to self‐management of their asthma (Fegran et al., 2016). Nonetheless, gaps exist in asthma education for youths, particularly those moving into adulthood (Rhee et al., 2009; Strof et al., 2012; Uzuncakmak & Beser, 2017). Many are ill prepared to independently manage their asthma (Kew et al., 2017; Uzuncakmak & Beser, 2017; Sleath et al., 2016). Their lack of asthma knowledge and the lack of empathy from healthcare providers have been indicated in adding to the anxiety of taking responsibility in the management of their asthma (Kew et al., 2017).

Studies of effectiveness of self‐management asthma education for youths, has demonstrated positive results, including improved symptom management and improvement in quality of life (Kew et al., 2017). Specifically, the Cochrane review provides evidence of peer and lay‐led asthma education with a focus on the empowerment of youths in their choices around their asthma health (Kew et al., 2017). Empowerment of youths through asthma self‐management education shifts the dynamic from didactic education to an inclusive, respectful peer partnership to attain asthma goals (Kew et al., 2017). However, there is lack of qualitative research specifically addressing youths' experiences of self‐management and asthma education (McTague et al., 2019).

The aim of this systematic review is to identify the available evidence, appraise and synthesize the findings from qualitative research that relates to youths and their experience of self‐management education. In addition, the synthesis of evidence from the youths' viewpoint will provide a foundation for future development of asthma educational interventions for healthcare providers seeking to meet the expressed needs of this cohort. Adopting the Joanna Briggs methodology for carrying out systematic reviews we aim to highlight implications for practice and policy, which will be informed by the quality of the included studies and the contexts in which the studies have been conducted.

## 2 THE REVIEW

### 2.1 Aims

This systematic review aimed to synthesize the best available qualitative evidence on youths with asthma and their experiences of self‐management education. The objectives were to:

- Explore how youths, with a diagnosis of asthma, experience asthma self‐management education.
- Highlight specific issues related to the self‐management of asthma for youths with this chronic condition.
- Present recommendations for research, education and the clinical context

### 2.2 Design

The systematic review was conducted using the meta‐aggregation approach in accordance with the JBI methodology for systematic reviews of qualitative evidence (Lockwood et al., 2020). It followed the methods established in the a priori protocol registered with PROSPERO CDR42019138083 (McTague et al., 2019). Papers were included or excluded based on the criteria set out below. The PRISMA 2020 statement reporting guidelines were used in preparing this manuscript (Appendix S1).

### 2.3 Search methods

#### 2.3.1 Inclusion/exclusion criteria

##### Participants

This review includes studies involving youths with a diagnosis of asthma who had experienced self‐management education. For this review, we adopted the UN definition of ‘youth’ “as those persons between the ages of 15 and 24 years, without prejudice to other definitions by Member States” (United Nations, 2013). If the age range of the studies was unclear or included those outside our age criteria, we excluded the study if the mean age was not between 15 and 24 years (Kew et al., 2017). There was no restriction on year of publication. Studies were also excluded if all participants were aged under 15 years or if insufficient data were provided to establish the mean age of participants. Where studies explored the experiences of parents and/or healthcare professionals as well as those of the youths, we extracted data solely related to youths' experiences of self‐management education.

##### Phenomenon of interest

Self‐management education as experienced by youths with a diagnosis of asthma.

##### Context

Studies exploring youths with asthma experiences of self‐management education in primary care, hospital and community settings (excluding schools) which include general practice, nurse‐led clinics, public health services and all hospital settings.

##### Types of studies

Studies that focused on qualitative data, including international studies published in English were considered for inclusion in this review. According to Clarke (2016), there is a need for systematic reviews. In line with our published protocol (McTague et al., 2019) and given the research aims, it was decided that qualitative data would provide the rich data on how youths with asthma experience self‐management education related to their condition. No date limit was set for the database searches.

### 2.4 Search strategy

The search strategy involved a comprehensive three‐phase process: (i) a search of academic databases for published studies, (ii) a search of sources of grey literature for unpublished studies and (iii) a hand search of reference lists for studies unidentified in the other two searches. Initial scoping searches using the database thesauri were run in CINAHL Complete, Embase, MEDLINE (EBSCO), ASSIA and PsycINFO, these searches provided a list of focused index terms and a list of synonyms. Search terms used included asthma, self‐management, education and qualitative research. Further Analysis of the keywords contained in the title and abstract, and of the index terms used to describe the articles retrieved during the search was used to create a comprehensive list of keywords. Six databases were selected for searching, CINAHL Complete (1937‐), Embase (1990‐), MEDLINE (Ovid) (1965‐), PsycINFO (1990‐), ASSIA and the Global Index Medicus. The search for unpublished or grey literature included Open Grey, Google Scholar, RIAN, LENUS, ProQuest Dissertations and Theses. Reference lists of included studies were reviewed to identify additional relevant studies.

No concept was created for ‘youths’ as best practice indicates that population by age is filtered at the title and abstract screening phase under include/exclude criteria. The searches were initially conducted in September 2019, updated in July 2020 and July 2022 (see Appendix S2).

### 2.5 Search outcomes

**FIGURE 1:**
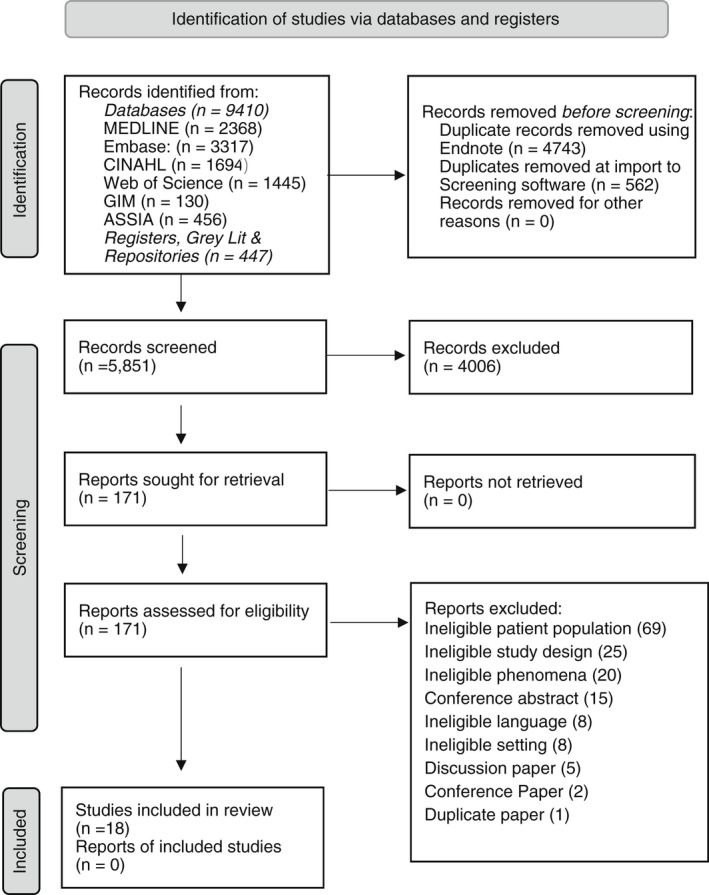
PRISMA flow diagram (Page et al., 2021)

Searches of academic databases and grey literature were carried out by the Subject Librarian involved in the review. A hand search of reference lists of retrieved papers for inclusion was completed by two of the reviewers. Figure 1 contains a diagrammatic representation of the search strategy that is based on PRISMA 2020 (Page et al., 2021).

The results were exported from each database and uploaded into Endnote X9 (Clarivate Analytics) and duplicates were removed (Bramer et al., 2016). Titles and abstracts were screened using the *Covidence* systematic review software (Veritas Health Innovation, 2021) by two independent reviewers for assessment against the inclusion criteria. Potentially relevant studies were retrieved for full screening. All studies were screened to full‐text review stage and then narrowed to the English language. Disagreements arising between the reviewers were resolved through discussion or with a third reviewer.

### 2.6 Quality and confidence appraisal

#### 2.6.1 Assessment of methodological quality

The quality of the papers was assessed using the JBI Critical Appraisal Checklist for Qualitative Research; a standardized critical appraisal instrument from the Joanna Briggs Institute System for the Unified Management, Assessment and Review of Information (JBI SUMARI) (Lockwood et al., 2020; Table 1). Two independent reviewers assessed qualitative papers selected for retrieval for methodological validity prior to inclusion in the review. Studies were excluded on the basis of not meeting the predefined eligibility criteria set out above. If the age of participants was not stated or if the mean age was not clear or could not be calculated, studies were excluded. Reviewers were blinded to each other's assessments; only after initial appraisal of each article had been completed by both reviewers could assessments be compared. Disagreements arising were resolved through discussion or with a third reviewer and the remaining studies are the final number included in this systematic review. The results for each study indicated moderate to high scores with 17 papers receiving a score of seven or more out of 10. Half of the studies (50%) had a statement locating the researcher culturally or theoretically and only 33% indicated the influence of the researcher on the research. No study received a low score indicating the need to exclude from the review.

**TABLE 1:** Critical appraisal results of eligible studies

Study	Q1	Q2	Q3	Q4	Q5	Q6	Q7	Q8	Q9	Q10
Buston and Wood (2000)	Y	Y	Y	Y	Y	U	N	Y	N	Y
Coombs et al. (2018)	Y	Y	Y	Y	Y	Y	N	Y	Y	Y
Davis et al. (2021)	Y	Y	Y	Y	Y	U	U	Y	Y	Y
Gibson‐Scipio et al. (2015)	Y	Y	Y	Y	Y	Y	U	Y	Y	Y
Heyduck et al. (2015)	Y	Y	Y	Y	Y	U	N	Y	Y	Y
Holley et al. (2018)	Y	U	Y	Y	U	U	N	Y	Y	Y
Hui et al. (2021)	Y	Y	Y	Y	Y	Y	Y	Y	Y	Y
Jonsson et al. (2017)	Y	Y	Y	Y	Y	Y	Y	Y	Y	Y
Koster et al. (2015)	Y	Y	Y	Y	Y	Y	U	Y	Y	Y
Milnes et al. (2013)	U	Y	Y	Y	Y	N	U	Y	Y	Y
Ödling et al. (2020)	Y	Y	Y	Y	Y	Y	U	Y	Y	Y
Peters et al. (2017)	Y	Y	Y	Y	Y	U	Y	N	Y	Y
Quaranta et al. (2014)	Y	Y	Y	Y	Y	N	N	N	Y	Y
Ramsey et al. (2019)	Y	Y	Y	Y	Y	U	N	N	Y	Y
Rhee et al. (2006)	Y	Y	Y	Y	Y	Y	Y	Y	Y	Y
Rhee, Allen, et al. (2014), Rhee, Fairbanks, et al. (2014)	Y	Y	Y	Y	Y	N	U	Y	Y	Y
Rydström et al. (2005)	Y	Y	Y	Y	Y	Y	U	Y	U	Y
Zaeh et al. (2021)	Y	Y	Y	Y	Y	Y	Y	Y	Y	Y
% Yes responses	94	94	100	100	94	50	28	83	89	100

*Note*: Y = yes, indicates a clear statement appears in the paper which directly answers the question; N = no, indicates the question has been directly answered in the negative in the paper; U = unclear, indicates there is no clear statement in the paper that answers the question or there is ambiguous information presented in the paper. Criteria for the critical appraisal of qualitative evidence: Q1: Is there congruity between the stated philosophical perspective and the research methodology? Q2: Is there congruity between the research methodology and the research question or objectives? Q3: Is there congruity between the research methodology and the methods used to collect data? Q4: Is there congruity between the research methodology and the representation and analysis of data? Q5: Is there congruity between the research methodology and the interpretation of results? Q6: Is there a statement locating the researcher culturally or theoretically? Q7: Is the influence of the researcher on the research, and vice versa, addressed? Q8: Are participants, and their voices, adequately represented? Q9: Is the research ethical according to current criteria or, for recent studies, is there evidence of ethical approval by an appropriate body? Q10: Do the conclusions drawn in the research report flow from the analysis, or interpretation, of the data?

Abbreviations: N, no; U, unclear; Y, yes.

### 2.7 Data abstraction and synthesis

**FIGURE 2:**
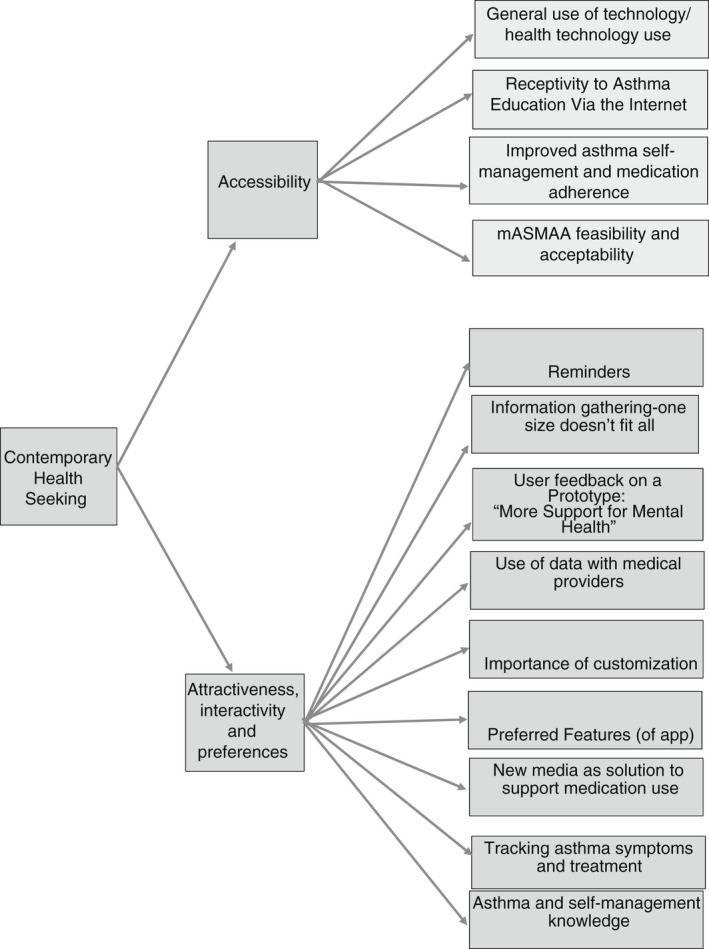
Example of meta‐aggregation process, themes and subthemes

Qualitative data were extracted by two independent reviewers guided by a recognized framework (Lockwood et al., 2020). This included specific details about the authors, year and country, phenomena of interest, participants, study methods, methodology and the main results of each study. The findings were then extracted using verbatim and non‐verbatim statements and were reviewed and pooled using the meta‐aggregation approach (Lockwood et al., 2020; Munn et al., 2014). A set of statements was generated to represent an aggregation of the findings. This involved collecting the findings and categorizing those findings based on a similarity in meaning. Following the principles of meta‐aggregation, findings were synthesized into themes. An example of the meta‐aggregation process is outlined in Figure 2.

## 3 RESULTS

### 3.1 Study characteristics

This review retrieved 9857 articles in total, following removal of duplicates, 5851 articles were screened on title and abstract by two reviewers. Following initial screening, 171 full‐text articles were screened in depth for suitability, of these, 153 were deemed unsuitable (see Prisma flow diagram). A final tally of 18 papers were eligible for inclusion in this systematic review.

Studies meeting the inclusion criteria were published after 2000 with the majority published after 2012. The geographical location and the number of studies were: the United States of America (*n* = 6), Sweden (*n* = 3), United Kingdom (*n* = 4), Australia (*n* = 3), Germany (*n* = 1) and the Netherlands (*n* = 1). All the studies used qualitative research methods that explored youths with asthma and their experience of self‐management education. The methods used to collect data include individual interviews, focus groups, participant observation and participatory workshop. A list of the papers (*n* = 18) and their key characteristics is contained in Table 2. As noted above, we extracted data solely related to youths' experiences of self‐management education.

**TABLE 2:** Characteristics of included studies

Study and country	Phenomena of interest	Participants	Methods	Methodology	Main results
Buston and Wood (2000) , United Kingdom	Examine treatment non‐compliance in adolescents with asthma	Adolescents (*n* = 49) aged 14–20 years Twenty males and 29 females	Individual interviews Thematic analysis	Qualitative description	Importance of adherence to prescribed medication plan. Main reasons for non‐compliance were forgetfulness, belief that medication was ineffective, denial about condition, difficulty using inhalers, inconvenience, fear of side effects and stigma
Coombs et al. (2018) , Australia	Explore youths' health literacy asthma experiences and health seeking behaviours	Youths (*n* = 20) aged 18–24 years Gender not reported	Individual interviews Interpretative phenomenology	Phenomenology	Issues regarding living with asthma, constant fear, reliance on parental care and support. Individualized nature and experiences of asthma and tailored supports and interventions
Davis et al. (2021), Australia	Understand the user experience and test the acceptance, engagement and effectiveness of an Asthma app	Young people aged 15–25 years (*n* = 9) Gender breakdown not specified	Thematic content analysis of open‐ended questions from a follow‐up survey	Qualitative descriptive	The app had many features the participants enjoyed. They reported the app was visually appealing and enable them to set goal, self‐monitor (logging and tracking) and set medication reminders. They also suggested additional functionality they would like to see on the app (check your inhaler technique) but removal of some superfluous features
Gibson‐Scipio et al. (2015) , USA	Explore the experiences of African American adolescents with asthma regarding transition to adult healthcare	Adolescents (*n* = 13) aged 14–18 years Six males and seven females	Focus groups Thematic analysis	Qualitative description	Key concerns around medication self‐management, social supports, independence versus interdependence, self‐advocacy. More guided support required with healthcare
Heyduck et al. (2015)^a^, Germany	Explore adolescents and caregivers perceptions of asthma self‐management	Adolescents (*n* = 15) aged 11–17 years Eight males and seven females	Focus groups Content analysis Dyadic analysis	Qualitative description	Beliefs and views about individual asthma management. Parental supportive role and responsibilities in asthma care versus the adolescents' perspective
Holley et al. (2018)^a^, United Kingdom	Identify barriers to self‐management in adolescents with asthma	Adolescents (*n* = 20) aged 12–18 years Six males and 14 females	Focus groups and interviews Thematic analysis	Qualitative description	Importance of routine, embarrassment and developing confidence, effective communication with practitioners to facilitate an open and inclusive two‐way consultation. Identified roles and responsibilities for adolescents, parents and healthcare professionals regarding asthma self‐management
Hui et al. (2021)^b^, United Kingdom	Explore the functionality, helpfulness and reliability of patients trust in an IOT (Internet of Trust) system to deliver components of self‐management	Young adults (*n* = 3) of a specific age bracket 16–25 years Gender breakdown not specified	In‐depth semi‐structured interviews Thematic analysis	Qualitative description	The participants found the IoT system to be helpful in supporting a broad range of self‐management tasks (Identifying unusual symptoms, alerts for improper inhaler techniques, detecting unusual use of rescue medication, setting reminders). However, they did express concerns about the reliability of the IoT systems, in generating novel advise or reach diagnostic conclusions, by interpretating their data
Jonsson et al. (2017) , Sweden	Explore the daily life experiences of adolescents with asthma	Adolescents (*n* = 10) aged 16–18 years Five males and five females	Individual interviews Systematic Text Condensation Analysis	Qualitative description	Four categories identified in the interviews in relation to daily experiences with asthma: *Insights and understanding, Asthma not the focus of daily life, being acknowledged and being affected by asthma symptoms* A desire to interact as “normal” people and therefore potentially ignoring symptoms
Koster et al. (2015)^a^, the Netherlands	Assess adolescents' needs and preferences regarding medication counselling and support, with the focus on new media	Adolescents (*n* = 21) aged 14–16 years Ten males and 11 females	Focus groups Online and traditional face‐to‐face focus groups Inductive thematic analysis	Qualitative description	Forgetting medication was a major reason for not using medication as prescribed or being indifferent about the perceived need or beliefs about the actual benefits of asthma medication. Parents bridged this gap, acting as the reminder to adolescents to take medication and collect prescriptions, however the benefit of smartphone applications and access to online information would be helpful to promote a move towards independent asthma self‐management incorporating the importance of medication adherence
Milnes et al. (2013)^b^, United Kingdom	Evaluate a pre‐consultation guide developed to promote young people's participation by increasing their confidence in communicating asthma symptoms and asking questions	Young people aged 14–18 years (*n* = 24) with asthma and the practice nurses (*n* = 9) with whom they consult Nine males and 15 females	Exit interviews 3 months post use of the pre‐consultation guide Framework approach	Qualitative description	Young people identified the most useful components of a pre‐consultation guide for asthma to be the peer written sections, including example questions to ask in a consultation; and prompts for reflection on the impact asthma had on everyday life. There are key aspects of the pre‐consultation guide such as peer written components and guidance regarding self‐assessment of symptoms that could be applicable to interventions for young people with other long‐term conditions
Ödling et al. (2020) , Sweden	Explore young adults with severe asthma experiences of transition from paediatric to adult healthcare	Adolescents (*n* = 16) aged 22–24 years Seven males and nine females	Semi‐structured interviews Thematic analysis	Qualitative description	Responsibility for asthma management lay with them, the ties had been severed with the security, supports and familiarity of the paediatric setting and this was daunting. They felt joint preparation for adult healthcare if allowed would take time but would be beneficial. They were less supported in the adult healthcare setting and the felt that their asthma received insufficient support and interactions were healthcare providers were impersonal
Peters et al. (2017) , Australia	Explore the experience, needs and ideas of young people with asthma to define requirements for an asthma app that would be engaging and effective at improving their well‐being	Young people with asthma (*n* = 20) aged 15–25 years Eight males and 12 females	Participatory workshop (*n* = 13) Completed a workbook (incl. Those who could not attend workshop) (*n* = 13; *n* = 7) Demographics and 6‐item asthma control questionnaire Thematic analysis	Qualitative description	A need to consider psychological factors in app development. Asthma app should promote adolescents' sense of autonomy, competence and relatedness Technology‐based mental health support required for young people with asthma
Quaranta et al. (2014)^c^, USA	Understand how self‐management behaviours of adolescents with asthma are influenced by the perceived expectations for self‐management behaviours from healthcare providers, school nurses, teachers, family and friends	Rural adolescents (*n* = 7) aged 13–17 years Five males and two females	Focus groups: Rural adolescents (*n* = 2) Thematic analysis	Qualitative description	The results from this study demonstrate the influence of the expectations for asthma self‐management by significant people in the adolescents' life. Except for taking their prescribed medications, no other behaviours were addressed by their healthcare provider, parents, friends or school nurse. The lack of expectation for other self‐management behaviours that are essential for asthma control, such as knowledge of asthma symptoms, trigger avoidance and when to seek help during an asthma attack may be a leading contributor for uncontrolled asthma. Asthma action plans, if consistently used by healthcare providers, parents and schools, can reinforce the expectation for behaviours that will result in good asthma outcomes
Ramsey et al. (2019)^b^, USA	Obtain a deeper understanding of adolescents' general and health technology uses and their perceptions of how health technology may be beneficial in improving asthma self‐management	Adolescents with asthma (*n* = 20) aged 13–18 years (and their parents) Ten males and 10 females	Interviews (*n* = 20) Chart review Thematic analysis	Qualitative description	Majority had personal smart phone All used one type of health technology Technology to enhance self‐management: Used to track asthma symptoms and treatments using mobile devices (e.g. reminders and medication management) Video conferencing. Digital action plan Customization and individualization preferable. Health providers have access to adolescent medical information. Adolescents require a different approach than children and older adults
Rhee et al. (2006) , USA	Explore the asthma learning needs and Internet use and preferences of adolescents with asthma	Adolescents with asthma (*n* = 19) aged 12–18 years Eight males and 11 females	Focus group (*n* = 6) Thematic analysis	Qualitative description	Participants admitted having a limited knowledge base – primarily focused on inhaler use. They agreed there was a need for education on asthma and its management. Receptive to the possibility of using the Internet to obtain information (although none in the older groups had previously used the Internet to locate information on asthma). A potential asthma website should be entertaining. Were concerned about trustworthiness and legitimacy of the source and preferred those sites offered by health professionals affiliated with a reputable healthcare institution (e.g. university health system). Websites should be teen‐friendly—use of entertaining features such as animation, cartoon characters, videos and lyrics. Format needs to stimulate and sustain adolescents' interest. Interested in the possibility of using websites for communicating with healthcare providers and receiving immediate feedback
Rhee, Allen, et al. (Rhee, Allen, et al., 2014), Rhee et al. Rhee, Fairbanks, and Butz., A. (2014)^a^, USA	Develop and evaluate the feasibility and acceptability of a comprehensive mobile phone‐based asthma self‐management aid for adolescents (mASMAA) that was designed to facilitate symptom monitoring, treatment adherence and adolescent–parent partnership	Adolescents (*n* = 15)—aged 13–17‐parent dyads participated in a 2‐week trial. Nine males and six females (adolescents)	Development of mASMAA (Adults or adolescents not included) Pilot‐testing of prototype Completion of six routine asthma‐diary questions (two in the morning and four at bedtime) and control medication reminders sent daily by mASMAA. Adolescents also encouraged to text self‐initiated messages related to asthma at least twice per day. Content analysis	Quantitative analysis of response rates to diary questions Qualitative description of focus group data	The majority of adolescents and parents agreed that mASMAA was an attractive and convenient option to facilitate asthma self‐management, having acknowledged the multiple benefits in promoting a sense of control, awareness of symptoms/triggers, treatment adherence and adolescent–parent partnerships. Some participants even noted an improved asthma condition during the study period. However, quantification of the positive effects of mASMAA and the long‐term sustainability of the system and its impact on asthma outcomes remains to be determined
Rydström et al. (2005) , Sweden	Provide a theoretical understanding of how teenagers with asthma manage their everyday life in relation to their disease	Teenagers with asthma (*n* = 23) aged 13–18 years Eleven males and 12 females	Participant observation (15 observations) Individual interviews (*n* = 32) Focus Groups (n = 3) Grounded theory‐open, selective and theoretical coding	Qualitative description	Teenagers struggle with not letting the disease get the upper hand over life. Three strategies are used to manage the disease: keeping distance from the disease The teens did not take their medications, did not go to the medical check‐ups and did not listen to caution from others. Disinterested in education lectures, looked in the other direction or left the room. 2 challenging the disease Testing limits and exerting themselves. 3 taking the disease into consideration. 4 knowing and listening to the body, seeing yourself and others as a resource and taking responsibility for your health. Boys kept the disease at distance while girls took it into consideration
Zaeh et al. (2021)^b^ America	Explore the effects of transitional challenges from adolescents to young adult on asthma controller medication adherence	Young adults (*n* = 7) Aged 18–30 Years Four females and three males	Semi‐structured interviews Thematic analysis	Qualitative description	Participants identified personal challenges affecting adherence – this included taking responsibility for their self‐management and their lack of preparedness, their lack of understand about their asthma and their desire to be a young person like their peers. This led to forgetting to take medication and issues around embarrassment about medication use. Additionally, the financial cost of medication needs to be considered for young people and extending the use of rescue medication in the community setting for emergency situations.

a Studies where mean age was not reported or was below 15 years; authors report all findings but did not use them in the meta‐aggregation.

b Studies where other participant types are included, only findings for youths presented in this review.

c Studies where individual participant ages were reported, and researchers calculated the mean age.

During the meta‐aggregation process, three main themes emerged: theory and practice gap, contemporary health seeking and psychosocial impacts of living with asthma. In addition, eight descriptive subthemes provide depth of youths' experience of asthma self‐management education. The following sections explores the emergent themes and subthemes (see Table 3).

**TABLE 3:** Main themes and subthemes

Main themes	Subthemes	Subthemes include…
Theory and practice gap	Interdependent roles and personal responsibilities	Role of youths and others (parents, peers and healthcare providers) in self‐management; perceived expectations of others
	Illness management and medication compliance	Medication adherence and avoidance; health literacy and understanding
	Inclusive and receptive communication	Difficulty in communication and/or lack of engagement with healthcare professionals; peer written information
	Barriers and enablers to asthma knowledge and self‐management	The identified barriers and enablers
Contemporary health seeking	Accessibility	General use of technology and receptiveness to asthma education via internet
	Attractiveness, interactivity and preferences	Youth's preferences for self‐management information
Psychosocial impacts of living with asthma	Emotional, psychological and social impacts of living with asthma	Burden of asthma on everyday life; facing challenges; life goals
	Networks of supports	Support from family, caregivers, healthcare providers and peers

### 3.2 Theory and practice gap

Youths' experience of asthma self‐management education is influenced by a wide range of sources, formal and informal, with specific asthma self‐management foci. From the youth's perspective, the practice of everyday living with asthma is problematic and does not always align to the theory of self‐management. Noteworthy too are youths accounts of experiential learning derived out of their reflection of living with asthma.

#### 3.2.1 Interdependent roles and personal responsibilities

Youths' difficulty with self‐management education is evidenced from the confusion they experience about their roles and responsibilities in bridging the practice‐theory self‐management gap. Youths living with parents and caregivers discuss moving towards greater responsibility in their disease self‐management but tend to remain under the watchful eye of parents in reminding them about their asthma regimes. Factors that highlight the juxtaposition of stakeholders, parents and healthcare professionals (HCPs) and the autonomy of youths in their disease self‐management are illustrated:

Just because you turn 18 and become an adult on paper, that doesn't mean that you are fully educated and mature enough to take on all that responsibility that it implies (Ödling et al., 2020, p. 5)

Autonomous independent living, for some youth, is not a predictor of personal responsibility. There remains a reliance on parental knowledge of their asthma to navigate flare ups and exacerbations.

I really get mum to manage it for me … I go ‘mum, do I take my inhaler now?’ ‘Yeah, take your inhaler now’ …. Because, well, mum is the only one that's really got experience with this so no one else really knows what to do (Coombs et al., 2018, p.182)

However, reflection and experience play's an important role in identifying asthma triggers and being prepared to take personal responsibility to minimize potential asthma attacks.

When I go over to my friend's house, she's got a bunch of cats, so I always make sure I grab my rescue inhaler before I go to her house to keep it in my pocket (Gibson‐Scipio et al., 2015, p. e58)

#### 3.2.2 Illness management and medication compliance

The emphasis on medication adherence, as the pillar of asthma self‐management, is replete in the studies included, however, arising out of the youth's illustrations an identifiable asthma medication theory‐practice gap exists. Youth's experiences focus on the task to “take medication” rather than possessing a sound knowledge base of ‘the what, why and when’ of self‐administration of their asthma medication, which is the corner stone of self‐management.

I try to work as hard as I can and not use my medicine as much so I won't have to depend on it, I try to use it (asthma medication) the least amount of times as I can so that I can get rid of the asthma and slow it down or something (Gibson‐Scipio et al., 2015, p. e57)

Self‐management or rather self‐taught education arises from experiential learning from “flare ups” of asthma symptoms and reflection on symptomatic events and often features in identifying the knowledge deficit of their medication regimes.

A month ago, I was really sick and coughed at night. I made a schedule for when to use the inhaler device, and then it worked better (Jonsson et al., 2017, p. 26)

#### 3.2.3 Inclusive and receptive communication

Youths' narrative of interactions with asthma self‐management stakeholders, illustrate concerns they have about not been heard, their voice and experiences being silenced, and their experience of one‐way communication, limiting the opportunity for youths to communicate asthma self‐management educational needs. Youths often experience a feeling of being “divorced” from healthcare consultations and often feel overshadowed by parents or caregivers.

Mum talks and I sit there and listen, but then I don't think the doctor fully knows how it's been for me, but mum always says I don't talk, but I would talk if I was given the chance to talk… I don't think they fully know cause when I come out, I think I would have said this and I would have said that but I didn't have the chance to (Holley et al., 2018, p. 949)

In supporting effective communication by youths with HCPs, there is evidence that pre‐ consultation aids and tools for communication is something that the youths felt would be useful to enable them to seek out information that was youth‐centred and specific to their needs.

When I go, I don't think about questions that I want to ask before I go. I always kind of think that it's always about the medication rather than how I feel, so I think if I read through some of these questions I'd be able to think about what to say…If I read this I'd get to think about more what I wanted to get from the asthma nurse rather than just inhalers (Milnes et al., 2013, p. 93)

#### 3.2.4 Barriers and enablers of asthma knowledge and self‐management

Barriers and enablers to self‐management reflect the uniqueness of youths in chronic disease self‐management. The constraints of the disease and the importance of routine and regime often provide the ideal breeding ground for conflict with a disease which youths struggle and battle against. Barriers are often constructed by the youths themselves in defiance and denial. Asthma is seen as an intruder in their lives.

The worst thing you can ask a teenager is to remember something again and again and again…and their gonna be like ‘oh I'm not gonna do this’ and just give up or something…they just won't bother…it sounds off giving someone who has no sense of responsibility something to be responsible about it (Holley et al., 2018, p. 949)

While barriers were identified, many youths acknowledge asthma as part of their lives. They have developed capacity for juggling the demands of being a youth with asthma, arming themselves with knowledge and ways of knowing as a positive outcome about living better with asthma.

It helped me … . the knowledge …. I think it's a lot of help when she explains something to me … it's made it a lot easier to manage cause I know what's happening (Holley et al., 2018, p. 948)

### 3.3 Contemporary health seeking

The buy‐in for contemporary health seeking as evidenced from the findings reveal a catalogue of requirements from accessibility, attractiveness to interactivity with options to adapt personal preferences. Youths identified the acceptability of using their phone as one said, “I always have my phone, my phone is‐this is my life” (Rhee, Allen, et al., 2014; Rhee, Fairbanks, et al., 2014, p. 67). Youths describe a willingness for tracking technology that may enable a growth in their confidence and autonomy in their disease management.

#### 3.3.1 Accessibility

Independent asthma self‐management is the goal of all stakeholders; however, the transition should be incremental to ensure confidence building in youths' capabilities to recognize and reflect on self‐management of their asthma. Technology can provide the accessibility of anytime anywhere support for youths and facilitate growth in their confidence and their role in disease self‐management.

I feel like it could help you manage your asthma because it like tells you when like you're having flare‐ups and what time of day and then that can help you find out why because you can reflect and think back what you were doing then or what you were exposed to (Rhee, Allen, et al., 2014; Rhee, Fairbanks, et al., 2014, p. 68)

#### 3.3.2 Attractiveness, interactivity and preferences

The issue of accessibility alone may not be sufficient for youths. Youths indicated they would like bespoke technological capabilities that could provide “like a digital asthma action plan that provides management instructions based on current, reported symptoms” (Ramsey et al., 2019, p. 968). They also suggested that technology could be used by their medial team to contact them about their condition.

It would be great if [the medical team] could reach out if they see something abnormal (Ramsey et al., 2019, p. 969)

The attractiveness, interactivity and preferential nuance of technology that appeals were proposed by youths.

Reading facts, and triggers, and things like that, would be cool. Even just simple things of how much is too much of your puffer? (Coombs et al., 2018, p.183)

Noteworthy too are youth's awareness and insight into informed decision making about trusted resources in e‐health technology.

### 3.4 Psychosocial impacts of living with asthma and support networks

The impact of living with asthma has affected youths, emotionally, psychologically and socially, at an important juncture in their growth and development. The burden of living with asthma at times can be overwhelming and youths recognize the importance of support networks, particularly from allied peers and family, to help them navigate the challenges of everyday living with asthma.

#### 3.4.1 Emotional, psychological and social impacts of asthma

The diagnosis of a medical condition brings with it many emotional reactions. It is evident that emotions such as anger, denial, grief and frustration are felt and the sense of “why me” are explicit. There is little insight into how emotions are worked through from the youths' perspective or if they are addressed in asthma self‐management interactions by healthcare providers.

[My doctor was] talking about my lungs and my lung function and they said it was getting poorer…I wasn't really taking my medication every day, so after one day when I saw how poor my lung function was, it kind of hit me in the head…oh I need to take these pills (Zaeh et al., 2021, p. 560).

The psychological impacts and unpredictable nature of their asthma, a sense of living in fear, adds to their stress and anxiety. Furthermore, the social constraints of asthma are problematic for youths. Testing their asthma “limits” in social situations was reported in one study (Rydström et al., 2005). Moreover, youths often struggle with other people's perception and lack of understanding of the disease, which for youths was perceived as shaming and embarrassing with implicit and explicit accounts, evident in many of the studies.

If you do have to [take medication] in public or in an event or something like that it can be a bit awkward at first because it is just not something that people typically see (Zaeh et al., 2021, p. 560)

#### 3.4.2 Network of supports

Reliable support networks are predominantly constructed from close friendships with peers that they spend time with in social, educational and sporting settings, as well as family members, especially those who themselves have experience of living with asthma. Whilst healthcare providers are seen as having a designated role in their asthma self‐management, it is the people, who youths interact with through daily interactions, that understand best the challenges and optics of life with asthma.

I tell my friends so that if I start to go into having an asthma attack, they'll know. I would want them to help me if I needed my inhaler or something (Gibson‐Scipio et al., 2015, p. e57)

## 4 DISCUSSION

Asthma is a chronic and potentially life‐threatening respiratory condition, with youths being identified as an at‐risk group (Strof et al., 2012). The population of interest, youths, young people and adolescents, has been discussed for over two decades in the literature reviewed in this systematic review. However, there is still a shortfall in addressing asthma self‐management needs and countries reporting qualitative research with this group. Asthma and allergies are on the increase in this population and therefore urgent action is needed to support youths achieve optimal health (Cevhertas et al., 2020).

The viewpoint of youths is key to understanding and improving their experience of asthma self‐management education. According to the youths in the studies reviewed, there is a significant gap in self‐management education that falls short of the holistic perspective of youths with an asthma diagnosis. In fact, we propose that some enablers of asthma self‐management education are self‐taught. In living their lives to the full, the evidence provided by youths in this review purports a specific focus on medication adherence and management (Buston & Wood, 2000; Koster et al., 2015). The gap between the theory of self‐management and practice of living with asthma has widened for youths. It is unclear if this gap has grown or it is rate of growth has accelerated from childhood through to youthhood (Coombs et al., 2018). Indeed, a recent systematic review on adherence to inhaled corticosteroids for asthma identified the continued fallibilities in successfully understanding and supporting young adults with the disease (Murphy et al., 2021). Notably, in the review, young people are referred to as outliers in attending primary care settings for their asthma care, and where medication non‐adherence is more evident (Murphy et al., 2021).

In addition, it is difficult to determine if an opportunity to stop, pause and reflect with youths on their asthma self‐management, when moving and transitioning to independent self‐care, is considered to bridge this theory and practice gap (Ödling et al., 2020). The lack of preparedness for this transition was explicit and a challenging experience. Adapting to the daily encounters of living with asthma included self‐advocacy and individual reflection (Gibson‐Scipio et al., 2015; Jonsson et al., 2017). Therefore, healthcare providers need to have the knowledge and skills to deliver on the evolving requirements of this cohort and strive to provide youth‐centric asthma self‐management education tailored to their needs and requirements. Specifically, recommendations for healthcare policymakers demonstrate the need for youth‐centric pathways for early intervention to equip this cohort with the necessary knowledge and skills as they transition from paediatric models of care. A range of supports, including a multidisciplinary and multiagency approach, provision of outreach services between practice and community settings and youth‐centric peer support are recommended.

The results of the review also highlight potential solutions in the delivery of asthma self‐management education. The report of positive experiences is presented by youths in respect of contemporary health‐seeking behaviours, notably the employment of technology in asthma self‐management. Technology is a feature of daily life in developed countries (Rhee, Allen, et al., 2014; Rhee, Fairbanks, et al., 2014) and is an acceptable conduit that youths in these studies show a willingness to embrace as part of their self‐management education needs. Furthermore, the construct of technological features was very important. Through developer engagement and interactions with youths, the buds of youth‐centric perspectives provide a glimpse into the possibilities of contemporary asthma self‐management education (Coombs et al., 2018; Hui et al., 2021; Peters et al., 2017; Ramsey et al., 2019; Rhee, Allen, et al., 2014; Rhee, Fairbanks, et al., 2014). Findings from this review suggest that optimizing the scope and reach of technology, for youths with asthma, has the potential to engage youths in asthma self‐management education, asthma monitoring and asthma support that is flexible to their needs and requirements.

Finally, one of the overwhelming findings of this review is the lack of a considered psychosocial and self‐care component of asthma self‐management education for youths. Youths describe the many challenges they face daily (Cheng et al., 2022; Peters et al., 2017). Their friends and peers are living a life, restriction‐free, compared with the youths unrelenting consciousness, of living with asthma that they must acknowledge (Gibson‐Scipio et al., 2015 *)*. This awareness is intertwined with the strive for independence and serves to magnify the burden of asthma. This alertness brings to the fore the emotional and psychosocial impact of asthma that perhaps as their asthma child‐self they had little insight too (Jonsson et al., 2017; Milnes et al., 2013; Zaeh et al., 2021). Furthermore, the lack of understanding by lay people, of the restrictions that asthma can place on them, was evident and a source of frustration for youths. The life‐threatening component of the disease is misunderstood and the normalizing of abnormal symptoms, is often trivialized (Jonsson et al., 2017). Furthermore, there was evidence from the youths that the structure of self‐management education addressed the physiological requirements of asthma but from the studies included in this review the evidence to support the psychosocial components of self‐management education are lacking. Priority for the psychosocial and self‐care components of asthma self‐management education is recommended as a finding of this review.

### 4.1 Limitations

This is the first review to aggregate the evidence to better understand the perspectives of youths and their experience of asthma self‐management education. The illustrations provided in this systematic review are rich and highlight several areas for future development of asthma self‐management education in terms of design, structure and delivery in the context of the community and healthcare setting. Furthermore, much of the research was related to younger teens so specific research on this youth cohort is warranted. The main limitation of this systematic review related to the identification of studies within the inclusion criteria, age of youths 15–24 years. Additionally, the transitional profile of this cohort suggests that youths' enrolment into studies maybe limited by youth's availability as they fall between the paediatric and adult healthcare models.

## 5 CONCLUSION

This study aimed to synthesize the evidence on youths with asthma and their experience of self‐management education. The findings of the review reveal that studies exploring their perceptions and experience of asthma self‐management are limited. The known risks associated with this cohort, who have a diagnosis of asthma, points to a requirement for further research concentrated on this population. Furthermore, the development of holistic, youth‐centric asthma self‐management education is needed. We encourage others to examine the potential benefits that a holistic approach to youth's asthma self‐management education could provide, and thereby build an evidence base for healthcare practitioners to improve youth asthma outcomes. This review has highlighted the gaps in self‐management education for youths and identified potential methods of delivering the same, to include psychosocial and emotional components so finally, we recommend that the wilderness, that lies between childhood asthma diagnosis and youthhood, needs to be cultivated with appropriate ongoing support and asthma education bespoke and inclusive for all.

## AUTHOR CONTRIBUTIONS

All authors have agreed on the final version and meet at least one of the following criteria (recommended by the ICMJE [http://www.icmje.org/recommendations/]):

- substantial contributions to conception and design, acquisition of data or analysis and interpretation of data;
- drafting the article or revising it critically for important intellectual content.

## CONFLICT OF INTEREST

The authors confirm there is no conflicts of interest.

### PEER REVIEW

The peer review history for this article is available at https://publons.com/publon/10.1111/jan.15459.

## Supporting information


Appendix S1

Appendix S2

## Data Availability

The data that support the findings of this study are available from the corresponding author upon reasonable request.
