# Supplementary material for: Youths with asthma and their experiences of self‐management education: A systematic review of qualitative evidence
Source: J Adv Nurs. 2022 Oct 14;78(12):3987–4002. doi: 10.1111/jan.15459 (PMC9827903; doi:10.1111/jan.15459)
Supplement: Supplementary file 2 — Appendix S2 [file JAN-78-3987-s001.docx]

**Appendix 2: Search Strategy and Sample Search History**

**Perceptions of self-management and educational interactions for adolescents and young adults with asthma: a systematic review of qualitative evidence.**

**Concept 1:**

**self-management and educational interactions**

**CINAHL:** (MH "Self Care+") OR (MH "Self Administration+")

**Medline:** (MH "Self Care") OR (MH "Self Medication") OR (MH "Self Administration")

**Embase:** 'self care'/exp OR 'self-directed learning'/exp OR 'self medication'/exp

**GIM:** ((tw:(self-administer* OR self-medicat* OR “self-treat*” OR self-manag* OR “self manage” OR self-car* OR “self caring” OR self-adhere* OR “self adhere*” OR “self-efficacy” OR “self efficacy” ))

**Web of Science: keywords only**

**ASSIA:** MAINSUBJECT.EXACT("Selfdirected learning") OR MAINSUBJECT.EXACT.EXPLODE("Selfmonitoring") OR MAINSUBJECT.EXACT("Selfefficacy") OR MAINSUBJECT.EXACT.EXPLODE("Selfmedication")

**Keywords:**

self-administer* OR “self administer*” OR self-medicat* OR “self medicat*” OR “self treat*” OR “self-treat*” OR self-manag* OR “self manag*” OR self-car* OR “self car*” OR self-adhere* OR “self adhere*” OR selfmanag* OR selftreat* OR “self-directed learning” OR “self directed learning” OR “self-directed education” OR “self–led learning” OR “self led learning” OR “independent learning” OR “self-efficacy” OR “self efficacy”

**(educat* OR train*) N3 (interact* OR program* OR setting* OR intervent* OR instruct* OR encounter* OR promot*)**

**Web oF Science Keywords:**

(self-administer* OR “self administer*” OR self-medicat* OR “self medicat*” OR “self treat*” OR “self-treat*” OR self-manag* OR “self manag*” OR self-car* OR “self car*” OR self-adhere* OR “self adhere*” OR selfmanag* OR selftreat* OR “self-directed learning” OR “self directed learning” OR “self-directed education” OR “self–led learning” OR “self led learning” OR “independent learning” OR “self-efficacy” OR “self efficacy”)

**OR**

**(train* OR educat*) NEAR/3 (interact* OR program* OR setting* OR intervent* OR instruct* OR encounter* OR promot*)**

**Embase keywords:** 'self administer*' OR 'self medicat*' OR 'self treat*' OR 'self-treat*' OR 'self manag*' OR 'self car*' OR 'self adhere*' OR selfmanag* OR selftreat* OR 'self-directed learning' OR 'self directed learning' OR 'self-directed education' OR 'self–led learning' OR 'self led learning' OR 'independent learning' OR 'self-efficacy' OR 'self efficacy'

**(train* OR educat*) NEAR/3 (interact* OR program* OR setting* OR intervent* OR instruct* OR encounter* OR promot*)**

**Concept 2: Asthma**

**Medline:** (MH Asthma+) OR (MH Asthma, Occupational) OR (MH Asthma, Exercise-Induced) OR (MH Asthma, Aspirin-Induced) OR (MH Status Asthmaticus)

**Embase:** 'asthma'/exp

**Web of Science: keywords only**

**CINAHL** (MH Asthma+)

**GIM:** (tw:(asthma))

**ASSIA:** MAINSUBJECT.EXACT.EXPLODE("Asthma")

**Keywords: asthma* OR** asthmatic*

**Concept 3: Qualitative studies**

**Medline:** (MH Empirical Research+) OR (MH Community-Based Participatory Research) OR (MH "Focus Groups") OR (MH "Interviews as Topic") OR (MH "Qualitative Research+")

**Embase:** 'qualitative research'/exp OR 'exploratory research'/exp OR 'grounded theory'/exp OR 'naturalistic inquiry'/exp OR 'participatory research'/exp OR 'interview'/exp

**Web of Science: keywords only**

**CINAHL:** (MH "Qualitative Studies+") OR (MH "Empirical Research") OR (MH "Case Control Studies+") (MH "Focus Groups") OR (MH "Interviews+") OR (MH "Narratives+") OR (MH "Observational Methods+") OR (MH "Self Report+")

**ASSIA:** MAINSUBJECT.EXACT.EXPLODE("Qualitative data") OR MAINSUBJECT.EXACT.EXPLODE("Qualitative methods") OR MAINSUBJECT.EXACT.EXPLODE("Qualitative research")

**World Health Organisation:**

**Keywords:** hermeneutic* OR “action research*” OR “empirical research*” OR ethnograph* OR phenomenolog* OR “grounded theor*” OR “feminist research*” OR “explanatory research*” OR “exploratory research*” OR “participatory research*” OR interview* OR “One-to-one” OR “interpretive research*” OR “emancipatory research*” OR “participant observation*” OR “observational stud*” OR “comparative stud*” OR “narrative research” OR “narrative analysis” OR “descriptive stud*” OR “descriptive research” OR qualitativ* OR “exploratory stud*” OR “literature review” OR “interpretative analys*” OR “case stud*” OR survey* OR “focus group*” OR “content analys*” OR “thematic analys*” OR “thematic coding” OR “observation* method*” OR “naturalistic inquir*” OR “group discussion*”

**Embase keywords:**

hermeneutic* OR 'action research*' OR 'empirical research*' OR ethnograph* OR phenomenolog* OR 'grounded theor*' OR 'feminist research*' OR 'explanatory research*' OR 'exploratory research*' OR 'participatory research*' OR interview* OR 'one to one' OR 'interpretive research*' OR 'emancipatory research*' OR 'participant observation*' OR 'observational stud*' OR 'comparative stud*' OR 'narrative research*' OR 'narrative analysis' OR 'descriptive stud*' OR 'descriptive research*' OR qualitativ* OR 'exploratory stud*' OR 'literature review'/exp OR 'literature review' OR 'interpretative analys*' OR 'case stud*' OR survey* OR 'focus group*' OR 'content analys*' OR 'thematic analys*' OR 'thematic coding' OR 'observation* method*' OR 'naturalistic inquir*' OR 'group discussion*'

**Results Table:**

|  | **Medline** | **Embase** | **Web of Science (run on title and topic)** |
| --- | --- | --- | --- |
| **Total** | 2,049 | 2818 | [1,569](http://apps.webofknowledge.com.elib.tcd.ie/summary.do?product=WOS&doc=1&qid=5&SID=D5STPHhyc6v2oPNvGVY&search_mode=CombineSearches&update_back2search_link_param=yes) |
|  |  |  |  |
|  | **CINAHL** | **World Health Organisation Library: GIM** | **ASSIA** |
| **Total** | 1157 | 114 | 432 |

**Sample - CINAHL Search History**

| **#** | **Query** | **Limiters/Expanders** | **Last Run Via** | **Results** |
| --- | --- | --- | --- | --- |
| S11 | S4 AND S7 AND S10 | Expanders - Apply equivalent subjects Search modes - Boolean/Phrase | Interface - EBSCOhost Research Databases Search Screen - Basic Search Database - CINAHL Complete | 1,157 |
| S10 | S8 OR S9 | Expanders - Apply equivalent subjects Search modes - Boolean/Phrase | Interface - EBSCOhost Research Databases Search Screen - Basic Search Database - CINAHL Complete | 1,344,554 |
| S9 | hermeneutic* OR “action research*” OR “empirical research*” OR ethnograph* OR phenomenolog* OR “grounded theor*” OR “feminist research*” OR “explanatory research*” OR “exploratory research*” OR “participatory research*” OR interview* OR “One-to-one” OR “interpretive research*” OR “emancipatory research*” OR “participant observation*” OR “observational stud*” OR “comparative stud*” OR “narrative research” OR “narrative analysis” OR “descriptive stud*” OR “descriptive research” OR qualitativ* OR “exploratory stud*” OR “literature review” OR “interpretative analys*” OR “case stud*” OR survey* OR “focus group*” OR “content analys*” OR “thematic analys*” OR “thematic coding” OR “observation* method*” OR “naturalistic inquir*” OR “group discussion*” | Expanders - Apply equivalent subjects Search modes - Boolean/Phrase | Interface - EBSCOhost Research Databases Search Screen - Basic Search Database - CINAHL Complete | 1,299,846 |
| S8 | (MH "Qualitative Studies+") OR (MH "Empirical Research") OR (MH "Case Control Studies+") (MH "Focus Groups") OR (MH "Interviews+") OR (MH "Narratives+") OR (MH "Observational Methods+") OR (MH "Self Report+") | Expanders - Apply equivalent subjects Search modes - Boolean/Phrase | Interface - EBSCOhost Research Databases Search Screen - Basic Search Database - CINAHL Complete | 410,613 |
| S7 | S5 OR S6 | Expanders - Apply equivalent subjects Search modes - Boolean/Phrase | Interface - EBSCOhost Research Databases Search Screen - Basic Search Database - CINAHL Complete | 47,722 |
| S6 | asthma* OR asthmatic* | Expanders - Apply equivalent subjects Search modes - Boolean/Phrase | Interface - EBSCOhost Research Databases Search Screen - Basic Search Database - CINAHL Complete | 47,722 |
| S5 | (MH Asthma+) | Expanders - Apply equivalent subjects Search modes - Boolean/Phrase | Interface - EBSCOhost Research Databases Search Screen - Basic Search Database - CINAHL Complete | 37,024 |
| S4 | S1 OR S2 OR S3 | Expanders - Apply equivalent subjects Search modes - Boolean/Phrase | Interface - EBSCOhost Research Databases Search Screen - Basic Search Database - CINAHL Complete | 220,411 |
| S3 | (educat* OR train*) N3 (interact* OR program* OR setting* OR intervent* OR instruct* OR encounter* OR promot*) | Expanders - Apply equivalent subjects Search modes - Boolean/Phrase | Interface - EBSCOhost Research Databases Search Screen - Basic Search Database - CINAHL Complete | 107,362 |
| S2 | self-administer* OR “self administer*” OR self-medicat* OR “self medicat*” OR “self treat*” OR “self-treat*” OR self-manag* OR “self manag*” OR self-car* OR “self car*” OR self-adhere* OR “self adhere*” OR selfmanag* OR selftreat* OR “self-directed learning” OR “self directed learning” OR “self-directed education” OR “self–led learning” OR “self led learning” OR “independent learning” OR “self-efficacy” OR “self efficacy” | Expanders - Apply equivalent subjects Search modes - Boolean/Phrase | Interface - EBSCOhost Research Databases Search Screen - Basic Search Database - CINAHL Complete | 114,156 |
| S1 | (MH "Self Care+") OR (MH "Self Administration+") | Expanders - Apply equivalent subjects Search modes - Boolean/Phrase | Interface - EBSCOhost Research Databases Search Screen - Basic Search Database - CINAHL Complete | 55,217 |

**CINAHL Search History – Rerun July 2022**

| **#** | **Query** | **Limiters/Expanders** | **Last Run Via** | **Results** |
| --- | --- | --- | --- | --- |
| S13 | S5 AND S8 AND S11 | Limiters - Published Date: 20200101-20221231  Expanders - Apply equivalent subjects  Search modes - Boolean/Phrase | Interface - EBSCOhost Research Databases  Search Screen - Advanced Search  Database - CINAHL Complete | 125 |
| S12 | S5 AND S8 AND S11 | Expanders - Apply equivalent subjects  Search modes - Boolean/Phrase | Interface - EBSCOhost Research Databases  Search Screen - Advanced Search  Database - CINAHL Complete | 1,177 |
| S11 | S9 OR S10 | Expanders - Apply equivalent subjects  Search modes - Boolean/Phrase | Interface - EBSCOhost Research Databases  Search Screen - Advanced Search  Database - CINAHL Complete | 1,479,446 |
| S10 | hermeneutic* OR “action research*” OR “empirical research*” OR ethnograph* OR phenomenolog* OR “grounded theor*” OR “feminist research*” OR “explanatory research*” OR “exploratory research*” OR “participatory research*” OR interview* OR “One-to-one” OR “interpretive research*” OR “emancipatory research*” OR “participant observation*” OR “observational stud*” OR “comparative stud*” OR “narrative research” OR “narrative analysis” OR “descriptive stud*” OR “descriptive research” OR qualitativ* OR “exploratory stud*” OR “literature review” OR “interpretative analys*” OR “case stud*” OR survey* OR “focus group*” OR “content analys*” OR “thematic analys*” OR “thematic coding” OR “observation* method*” OR “naturalistic inquir*” OR “group discussion*” | Expanders - Apply equivalent subjects  Search modes - Boolean/Phrase | Interface - EBSCOhost Research Databases  Search Screen - Advanced Search  Database - CINAHL Complete | 1,434,621 |
| S9 | (MH "Qualitative Studies+") OR (MH "Empirical Research") OR (MH "Case Control Studies+") (MH "Focus Groups") OR (MH "Interviews+") OR (MH "Narratives+") OR (MH "Observational Methods+") OR (MH "Self Report+") | Expanders - Apply equivalent subjects  Search modes - Boolean/Phrase | Interface - EBSCOhost Research Databases  Search Screen - Advanced Search  Database - CINAHL Complete | 402,182 |
| S8 | S6 OR S7 | Expanders - Apply equivalent subjects  Search modes - Boolean/Phrase | Interface - EBSCOhost Research Databases  Search Screen - Advanced Search  Database - CINAHL Complete | 49,876 |
| S7 | asthma* OR asthmatic* | Expanders - Apply equivalent subjects  Search modes - Boolean/Phrase | Interface - EBSCOhost Research Databases  Search Screen - Advanced Search  Database - CINAHL Complete | 49,876 |
| S6 | (MH Asthma+) | Expanders - Apply equivalent subjects  Search modes - Boolean/Phrase | Interface - EBSCOhost Research Databases  Search Screen - Advanced Search  Database - CINAHL Complete | 38,053 |
| S5 | S3 OR S4 | Expanders - Apply equivalent subjects  Search modes - Boolean/Phrase | Interface - EBSCOhost Research Databases  Search Screen - Advanced Search  Database - CINAHL Complete | 236,126 |
| S4 | (educat* OR train*) N3 (interact* OR program* OR setting* OR intervent* OR instruct* OR encounter* OR promot*) | Expanders - Apply equivalent subjects  Search modes - Boolean/Phrase | Interface - EBSCOhost Research Databases  Search Screen - Advanced Search  Database - CINAHL Complete | 113,975 |
| S3 | S1 OR S2 | Expanders - Apply equivalent subjects  Search modes - Boolean/Phrase | Interface - EBSCOhost Research Databases  Search Screen - Advanced Search  Database - CINAHL Complete | 132,153 |
| S2 | self-administer* OR “self administer*” OR self-medicat* OR “self medicat*” OR “self treat*” OR “self-treat*” OR self-manag* OR “self manag*” OR self-car* OR “self car*” OR self-adhere* OR “self adhere*” OR selfmanag* OR selftreat* OR “self-directed learning” OR “self directed learning” OR “self-directed education” OR “self–led learning” OR “self led learning” OR “independent learning” OR “self-efficacy” OR “self efficacy” | Expanders - Apply equivalent subjects  Search modes - Boolean/Phrase | Interface - EBSCOhost Research Databases  Search Screen - Advanced Search  Database - CINAHL Complete | 123,431 |
| S1 | (MH "Self Care+") OR (MH "Self Administration+") | Expanders - Apply equivalent subjects  Search modes - Boolean/Phrase | Interface - EBSCOhost Research Databases  Search Screen - Basic Search  Database - CINAHL Complete | 58,261 |
